# Time use, unpaid care work, and income: a nationwide cross-sectional web survey of gender gaps among hospital physicians in Japan

**DOI:** 10.1186/s12913-026-14627-7

**Published:** 2026-05-20

**Authors:** Kae Okoshi, Yukinari Tokoro, Kayo Fukami, Koya Hida, Ryosuke Mizuno, Yosuke Yamamoto, Kazutaka Obama

**Affiliations:** 1 Department of Surgery, Japan Baptist Hospital, Kyoto, Japan; 2 Department of Surgery, Graduate School of Medicine, Kyoto University, 54 Shogoin-Kawahara-cho, Sakyo-ku, Kyoto, 606-8507 Japan; 3 Department of Medical Life Systems, Faculty of Life and Medical Sciences, Doshisha University, Kyoto, Japan; 4 General Education, National Institute of Technology, Toba College, Mie, Japan; 5 Department of Healthcare Epidemiology, Graduate School of Medicine, Kyoto University, Kyoto, Japan

**Keywords:** Gender, Physicians, Unpaid care work, Time use, Income, Workforce, Japan, Cross-sectional survey

## Abstract

**Background:**

Time is the most limited resource in medicine. When unpaid care and domestic work are distributed unevenly by gender, this can alter how physicians work, train, and earn—an international pattern with implications for equity and workforce sustainability. Here, we investigated gender differences in income and daily time allocation for paid work, unpaid care work (housework and child care), and other essential activities.

**Methods:**

We conducted a cross-sectional web survey of physicians registered with a large Japanese medical portal (m3.com) from January 9 to 31, 2024. Participants were full-time hospital physicians who met the inclusion criteria (*n* = 2,540; 2,224 men and 316 women). The exposure was physician gender (women vs. men). Outcomes were self-reported daily hours across seven time-use domains—unpaid care work on weekdays and on weekends/holidays (primary), paid work, academic and professional development, commuting, meals and personal care, leisure, and sleep—and personal annual income (≥ ¥15 million vs. < ¥15 million). We estimated adjusted gender differences in time use and the adjusted prevalence ratio for earning ≥ ¥15 million, controlling for key demographic and occupational factors.

**Results:**

Response and participation rates could not be calculated because the survey vendor did not disclose the sampling denominator. After multivariable adjustment, women spent more hours on unpaid care work than men on both weekdays (1.51 h/day; 95% confidence interval [CI] 1.31 to 1.70) and weekends/holidays (2.35 h/day; 95% CI 1.98 to 2.72). Women also reported fewer hours in paid work, academic and professional development, and leisure. Women were less likely than men to earn ≥ ¥15 million annually (adjusted prevalence ratio 0.65; 95% CI 0.56 to 0.76).

**Conclusions:**

In this cross-sectional sample of full-time hospital physicians in Japan, substantial gender disparities in income and time use remained after adjustment for major demographic and occupational factors. Universal caregiver supports should be designed to encourage and facilitate men’s participation, to avoid reinforcing gendered divisions of labor.

**Supplementary Information:**

The online version contains supplementary material available at 10.1186/s12913-026-14627-7.

## Background

Physicians’ long working hours are a global concern for well-being, patient safety, and workforce sustainability. While policies target paid hours to address this concern, evaluating their fairness requires tracking what happens to time outside work. In many societies, unpaid care and domestic labor remains unequally distributed by gender; thus, reductions in paid hours may not narrow these gaps.

Gender role theory holds that societal norms prescribe distinct roles for men and women [1], and work-family conflict theory highlights tensions between work and domestic demands [2]. International evidence further suggests that reducing men’s work hours alone may not eliminate disparities in unpaid care and domestic work: For example, time gained through flexible arrangements or parental leave can be diverted to leisure rather than childcare [3], and even when couples have comparable work hours, women perform more domestic work on days off while men engage more in leisure [4]. These findings underscore the need to track how much physicians work and how time is reallocated outside the workplace.

Despite growing policy attention, physician time-use data remain scarce and vary widely across countries and health systems. Where such data exist, male physicians’ participation in unpaid care and domestic work is generally limited and concentrated in financial tasks rather than routine housework or childcare [5]. In this context of limited evidence on physicians’ time use and persistent gender gaps in unpaid care and domestic work, Japan offers a timely case study. The Japanese health system has long relied on extensive physician overtime [6]. In 2019, Japan enacted work-style reform legislation that introduced overtime limits for many workers, mandated paid leave use, and promoted flexible work arrangements [7]. Under this framework, physician-specific hour-cap regulations were scheduled to take effect in April 2024. Structural gender inequalities have been documented among Japanese clinicians. For example, women surgeons report stronger prioritization of family responsibilities over career advancement [8], and during the COVID-19 pandemic, women physicians experienced disproportionate increases in domestic burdens [9].

Using data from a nationwide survey of physicians in Japan conducted in January 2024, 3 months before nationwide hour-cap regulations took effect, we aimed to quantify gender differences in daily time allocation across paid work, academic and professional development, unpaid care work (housework and child care), leisure, commuting, and sleep on weekdays and weekends. We hypothesized that, even at lower levels of paid hours, men would not proportionally increase unpaid care work, sustaining gender gaps in this domain. These estimates provide a pre-implementation baseline for how physicians allocate time within and beyond paid work. We define a simple set of mutually exclusive daily activity categories and estimate gender differences in hours per day for each category, an approach that can be directly replicated in other health systems to evaluate how hour caps affect unpaid care work, leisure, and other daily activities.

## Methods

### Study design, setting, and participants

We conducted a nationwide, cross-sectional, self-administered, anonymous web survey of physicians in Japan from January 9 to 31, 2024. This study followed the Strengthening the Reporting of Observational Studies in Epidemiology (STROBE) and the best practices of the American Association for Public Opinion Research (AAPOR). The sample size was determined by the constraints of the fixed fielding window and the available online panel; therefore, no a priori sample size calculation was performed.

The target population was full-time hospital physicians (defined as ≥ 32 h/week); physicians working < 32 h/week were considered part-time and were excluded. Only authenticated m3.com physician accounts could access the questionnaire; the platform allowed only one submission per account. Completion incentives consisted of standard m3.com points.

Given that invitation, view, and click-through denominators were not available to the investigators from the survey platform, formal AAPOR response/participation rates (RR1–RR6) could not be calculated. Accordingly, we report only the number of completed questionnaires and the field dates in the Results, and provide the AAPOR Survey Disclosure Statement in Additional file 1.

### Measures (exposures, outcomes, and covariates)

The questionnaire (Additional file 2) collected gender (male, female, or other), age, employment type, parental/caregiving leave, annual income, marital status, spouse’s occupation, presence and ages of children, and gender-role attitudes.

Respondents reported average daily time use for the most recent week, separately for weekdays and weekends/holidays, across working hours, commuting, academic/professional development (including conferences and research), unpaid care work, leisure, sleep, and meals and personal care. Self-reported time-use entries were constrained to whole hours that summed to 24 per day; therefore, we interpret estimated differences as reallocations of time across activity categories.

The primary outcome was hours/day of unpaid care on weekdays and on weekends/holidays. Secondary outcomes were hours/day for other time-use categories and annual income (≥ ¥15 million vs. < ¥15 million). Annual income was collected in multiple ordered categories and dichotomized for analysis at ¥15 million (≥ ¥15 million vs. < ¥15 million). The ¥15 million threshold was selected to provide a pragmatically interpretable split between higher- and lower-income groups and to ensure adequate numbers in both groups.

Covariates (selected a priori for conceptual relevance) included age, marital status, youngest child’s age, specialty, and working hours (working hours excluded when modeling working hours as an outcome). Specialty was grouped as internal medicine, surgery, other (including pediatrics, psychiatry, anesthesiology, and radiology), and early residents.

Age and working hours were modeled as continuous variables; the youngest child’s age was modeled as categorical to allow for nonlinearity.

### Data integrity and measurement constraints

Survey responses were obtained from an online physician panel. The platform was designed to allow only one submission per approved physician account, and responses were collected anonymously. Time-use entries were self-reported in whole-hour increments constrained to sum to 24 h per day.

### Inclusion and exclusion criteria

The analytic sample was restricted to full-time hospital physicians (≥ 32 h/week) with complete time-use data for both weekdays and weekends/holidays. We excluded respondents who were on temporary leave; were aged ≤ 24 or ≥ 90 years; had an implausible age difference with the youngest child (< 20 or > 61 years); reported commuting > 6 h/day; or reported total daily time > 24 h. Respondents working < 32 h/week were considered part-time and were excluded from analyses, as specified in Sect.  2.1. Gender was self-reported; “other” responses were excluded due to small numbers and internal inconsistency. In Japan, physicians typically graduate from medical school and obtain a license at 24 years of age at the earliest; therefore, respondents aged < 24 years were considered unlikely to have completed full physician training.

### Statistical analysis

Statistical analyses were conducted separately for weekdays and weekends/holidays. Time-use outcomes were modeled using multivariable linear regression, reporting adjusted mean differences (female–male) with 95% confidence intervals (CIs), adjusted for age, marital status, youngest child’s age, specialty, and working hours (hours omitted when modeling working hours). For the primary unpaid care work outcomes, we visually examined Q-Q plots of model residuals to assess major departures from normality. Because daily totals summed to 24 h, estimated differences from multivariable models were interpreted as reallocations of time across activity categories conditional on covariates. Adjusted mean hours by gender were obtained via marginal standardization (Stata margins), averaging over the observed covariate distribution; 95% CIs used the delta method. Income (≥ ¥15 million) was modeled using modified Poisson regression with robust (Huber–White) standard errors to estimate adjusted prevalence ratios (aPRs). Because no values were missing in the analytic variables, all analyses used complete-case data. Given the nonprobability, volunteer online panel, no sampling weights were applied. To assess the influence of family-structure variables, we conducted sensitivity analyses for weekday and weekend/holiday unpaid care work after excluding marital status and child-related variables from the models. In addition, a post hoc descriptive subgroup analysis was conducted among dual-earner parents. Statistical analyses were performed using Stata version 18.0 (StataCorp LLC, College Station, TX, USA).

### Data visualization

Daily time budgets were displayed as composition plots (stacked bars) by gender and day type; adjusted gender differences (female–male) with 95% CIs were shown as dot-and-whisker plots. Figures were generated in Stata and R (ggplot2) from model-based estimates.

### Ethical considerations

This study complied with the Declaration of Helsinki and Japan’s Ethical Guidelines for Medical and Health Research Involving Human Subjects. Participants provided electronic informed consent prior to survey completion. The protocol was approved by the Kyoto University Graduate School of Medicine Ethics Committee (R4069).

### Reporting guideline

This cross-sectional study followed the STROBE reporting guidelines.

## Results

### Participant characteristics

**Fig. 1:**
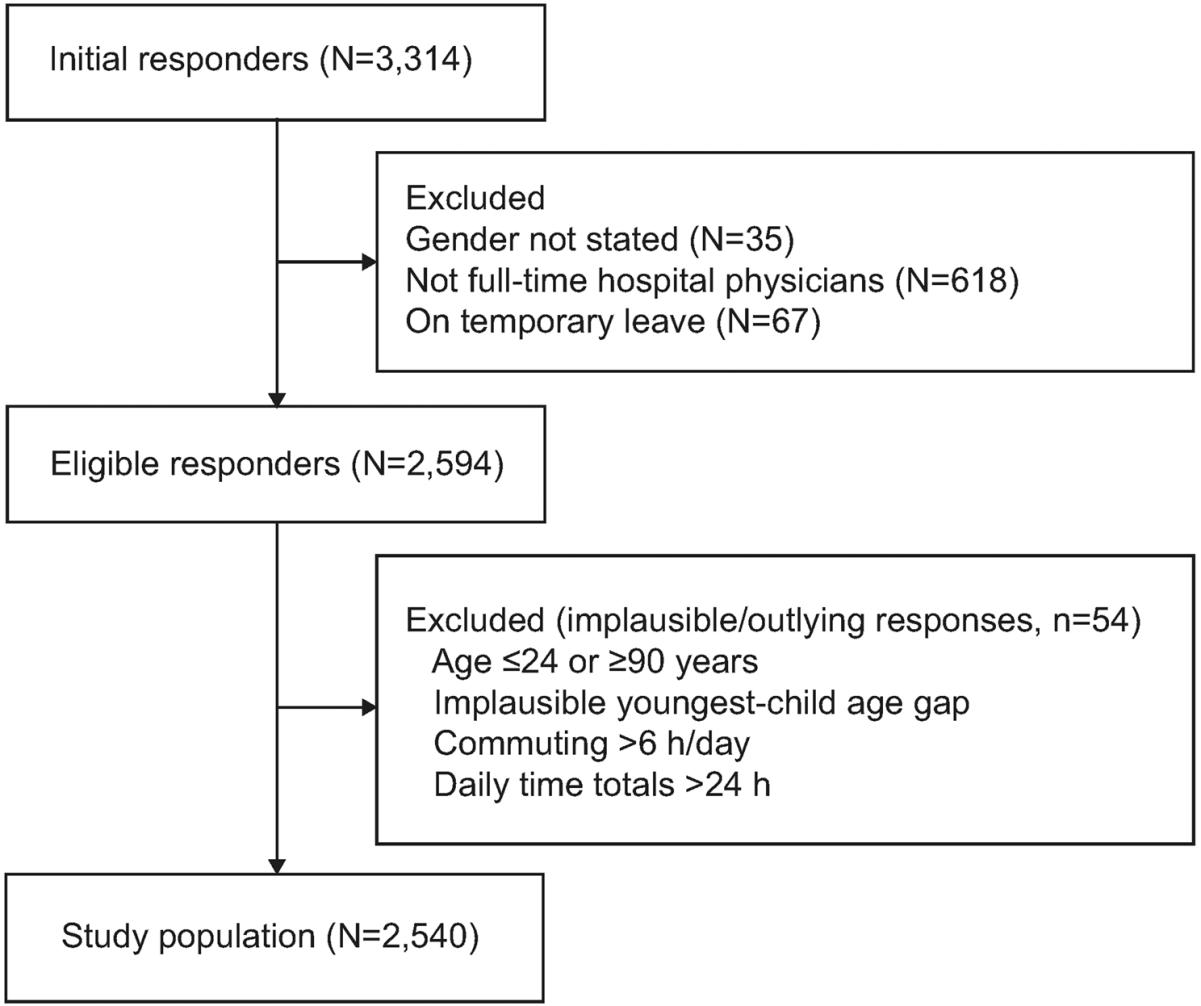
Participant flow diagram. Initial respondents (*N* = 3,314). Excluded: gender not stated (*n* = 35), not full-time hospital physicians (*n* = 618), on temporary leave (*n* = 67). Eligible respondents: *n* = 2,594. Further excluded for implausible or outlying responses (age ≤ 24 or ≥ 90 years, implausible youngest-child age gap, commuting > 6 h/day, or daily time totals > 24 h) (*n* = 54). Final analytic sample (*n* = 2,540)

Of 3,314 respondents, 2,540 met all inclusion criteria (2,224 men; 316 women) and were analyzed (Fig. 1). Respondents were excluded if they did not meet the definition of full-time hospital physicians, were on temporary leave, were aged ≤ 24 or ≥ 90 years, had an implausible age difference with the youngest child (< 20 or > 61 years), reported commuting > 6 h/day, or reported total daily time > 24 h.

Table 1 summarizes participant characteristics. Men were older on average (53.2 vs. 45.5 years) and more often married (89.5% vs. 58.5%). Men were less likely to have hospital-based physician spouses (11.6% vs. 29.4%). Higher income (≥ ¥15 million) was more common among men (62.8% vs. 34.2%).

**Table 1:** Background characteristics of survey respondents

Variable	Male (*n* = 2,224)	Female (*n* = 316)
Age, years, mean (SD)	53.2 (11.3)	45.5 (10.5)
Medical specialty, No. (%)		
Internal medicine	823 (37.0%)	105 (33.2%)
Surgeon	777 (34.9%)	93 (29.4%)
Other specialty	624 (28.1%)	118 (37.3%)
Marital status, No. (%)		
Single	234 (10.5%)	131 (41.5%)
Married	1990 (89.5%)	185 (58.5%)
Spouse/partner’s occupation, No. (%)		
Hospital-based doctor	259 (11.6%)	93 (29.4%)
Private practice doctor	15 (0.7%)	11 (3.5%)
Health care professionals (excluding doctors)	326 (14.7%)	7 (2.2%)
Office worker	74 (3.3%)	38 (12.0%)
Self-employed	25 (1.1%)	10 (3.2%)
Full-time homemaker	1072 (48.2%)	8 (2.5%)
Not working	67 (3.0%)	16 (5.1%)
Other	154 (6.9%)	2 (0.6%)
Personal annual income, No. (%)		
≤ ¥15 million (JPY)	827 (37.2%)	208 (65.8%)
≥ ¥15 million (JPY)	1397 (62.8%)	108 (34.2%)
Youngest child’s age, No. (%)		
No children	708 (31.8%)	167 (52.8%)
Children under 6 years old	314 (14.1%)	46 (14.6%)
Children aged 6–22 years	786 (35.3%)	80 (25.3%)
Children aged 23 or older	416 (18.7%)	23 (7.3%)

Data are No. (%) unless otherwise indicated. Age is presented as mean (SD). “Other specialty” includes dermatology, pediatrics, psychiatry, anesthesiology, radiology, and early-stage residents (postgraduate year [PGY] 1–2) not yet assigned a specialty. Income categories are reported in Japanese yen (JPY): < ¥15 million and ≥ ¥15 million. “Children aged 6–22 years” includes compulsory education through university age. Percentages may not sum to 100% because of rounding. Abbreviations: SD, standard deviation

### Primary outcome: unpaid care (reallocation of daily time)

**Fig. 2:**
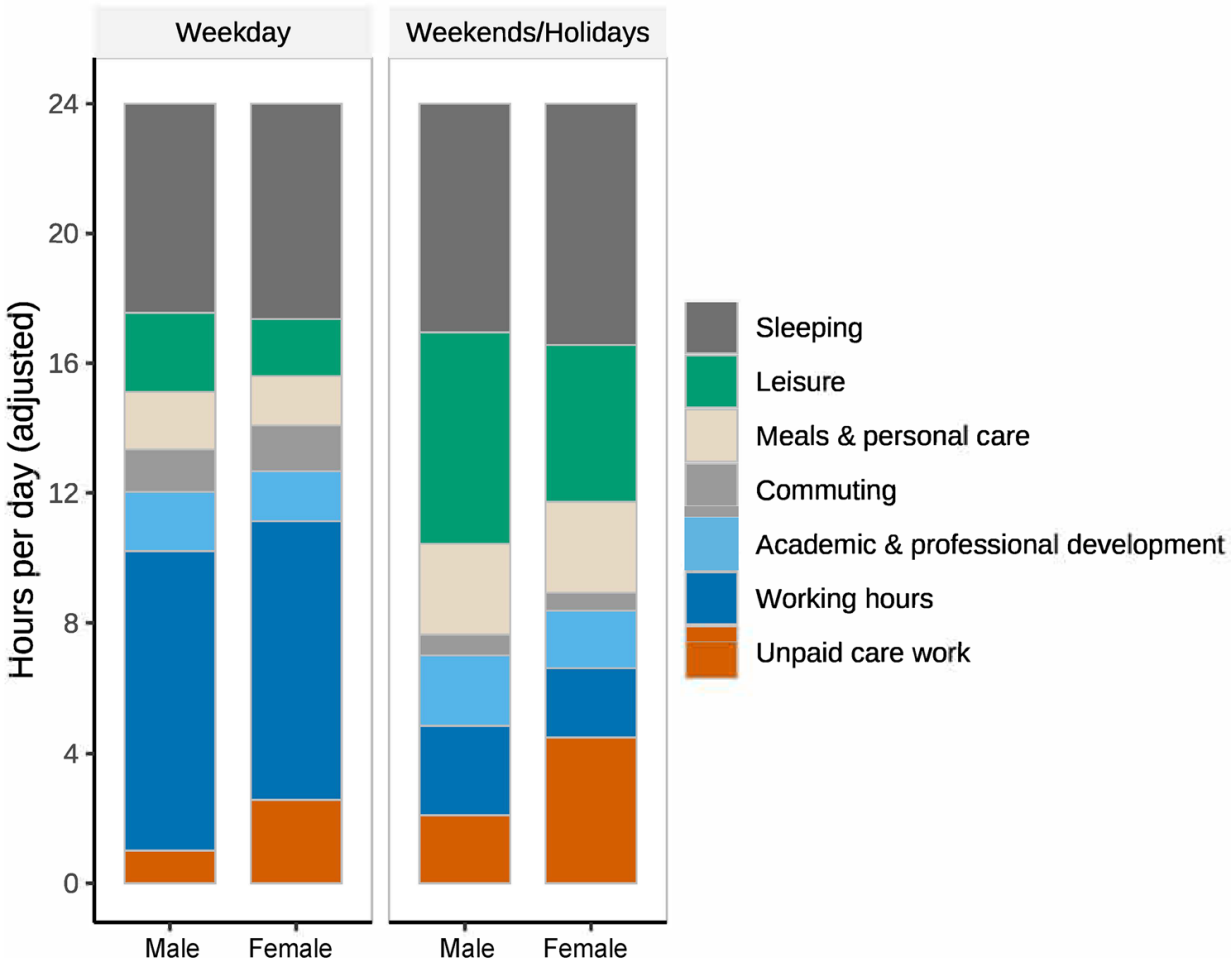
Adjusted 24-hour composition of time use by gender (weekdays vs. weekends/holidays). Stacked bars show adjusted mean hours/day across seven activity categories for men and women on weekdays and weekends/holidays. Estimates are from multivariable linear models adjusted for age, marital status, youngest child’s age, specialty, and working hours (working hours omitted when modeling working hours). Categories for each day sum to 24 h by construction

**Fig. 3:**
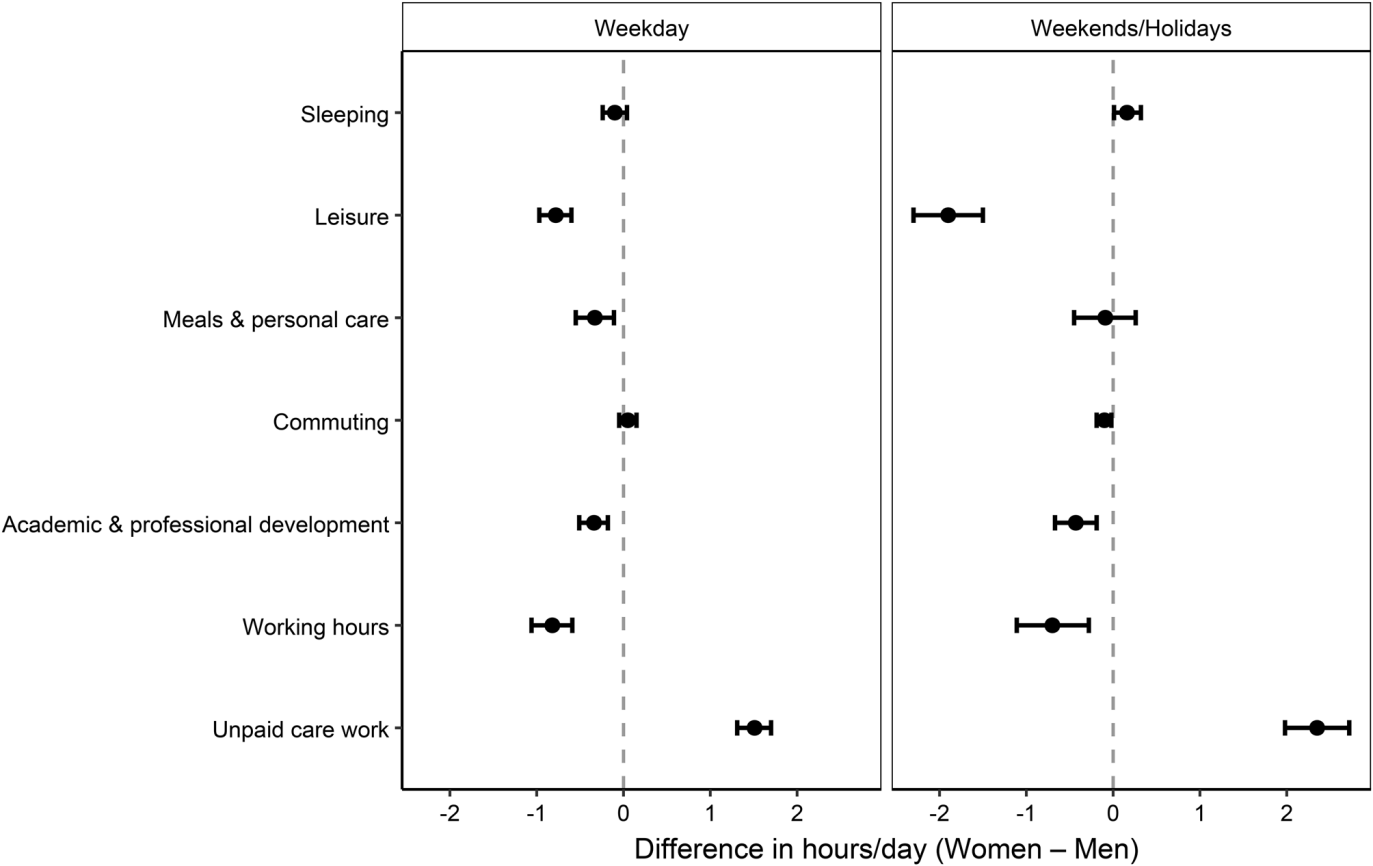
Adjusted gender differences in time use (female–male) with 95% CIs. Points show adjusted mean differences in hours/day (female minus male) with 95% CIs; separate panels display weekdays and weekends/holidays. Models adjust for age, marital status, youngest child’s age, specialty, and working hours (working hours omitted when modeling working hours). Positive values indicate more time for women; negative values indicate more time for men. Abbreviations: CI, confidence interval

Unadjusted time-use summaries by gender (hours/day) are shown in an additional table (Additional file 3). Figure 2 shows adjusted mean hours/day by gender for weekdays and weekends/holidays; Fig. 3 shows adjusted female–male differences with 95% CIs. Women spent more time on unpaid care than men on weekdays (+ 1.51 h/day; 95% CI, 1.31 to 1.70) and weekends/holidays (+ 2.35 h/day; 95% CI, 1.98 to 2.72) (Additional file 4, 5). In sensitivity analyses excluding marital status and child-related variables, the association between gender and unpaid care work remained statistically significant and in the same direction for both weekdays and weekends/holidays, although the magnitude of the coefficients was attenuated (Additional file 6).

### Other time-use domains

On weekdays, women reported less leisure (adjusted mean difference [AMD], − 0.78 h/day; 95% CI, − 0.97 to − 0.60) and less academic/professional development (AMD, − 0.34 h/day; 95% CI, − 0.51 to − 0.18), while commuting and sleep differences were small (commuting: AMD, 0.05 h/day; 95% CI, − 0.05 to 0.15; sleep: AMD, − 0.10 h/day; 95% CI, − 0.24 to 0.04). On weekends/holidays, women devoted less to leisure (AMD, − 1.90 h/day; 95% CI, − 2.30 to − 1.50), with slightly longer sleep (AMD, 0.16 h/day; 95% CI, 0.01 to 0.32) (Fig. 3; Additional files 4, 5).

### Weekday working hours

In multivariable linear regression, women worked fewer paid hours than men on weekdays (adjusted mean difference, − 0.82 h/day; 95% CI, − 1.06 to − 0.59) (Table 2). Differences by age were small (40–59 and ≥ 60 vs. ≤ 39 years), and specialty contrasts were minimal; marital status and the youngest child’s age were not clearly associated with weekday working hours (Table 2).

**Table 2:** Factors associated with weekday working hours (multivariable linear regression)

Variables	Adjusted mean difference, hours/day (vs. reference)	95% CI
**Gender**		
Male (reference)	—	
Female	−0.82	−1.06, − 0.59
**Age**		
≤ 39 years (reference)	—	
40–59 years	−0.27	−0.40, − 0.14
≥ 60 years	−0.28	−0.43, − 0.12
**Marital Status**		
Unmarried (reference)	—	
Married	0.01	−0.12, 0.10
**Specialty**		
Internal Medicine (reference)	—	
Surgeon	0.10	−0.08, 0.28
Other Specialty	−0.20	−0.38, − 0.02
**Youngest Child’s Age**		
≤ 5 years (reference)	—	
6–22 years	0.09	−0.17, 0.35
≥ 23 years	0.02	−0.31, 0.35
No Children	−0.10	−0.37, 0.17

Linear regression adjusted for gender, age group, marital status, specialty, and youngest child’s age. Reference categories are male, age ≤ 39 years, unmarried, and internal medicine. Negative values indicate fewer hours/day than the reference group. Abbreviations: CI, confidence interval

Taken together, the working-hours model suggests only modest between-group differences relative to the larger reallocations observed in unpaid care.

### Personal annual income

Unadjusted prevalence of annual income ≥ ¥15 million was 34.2% among women and 62.8% among men (Table 1) (crude prevalence ratio, 0.54; 95% CI, 0.47 to 0.64). After multivariable adjustment, women remained less likely than men to report annual income ≥ ¥15 million (aPR, 0.65; 95% CI, 0.56 to 0.76). Older age, being married, longer working hours, and being in surgery (vs. internal medicine) were associated with a higher prevalence of income ≥ ¥15 million, whereas youngest child’s age showed no clear associations (Table 3).

**Table 3:** Factors associated with higher individual income (≥¥15 million): adjusted prevalence ratios (aPR)

	Multivariable analysis	
	aPR	95% CI
**Gender**		
Male (reference)	—	
Female	0.65	0.56, 0.76
**Age**		
≤ 39 years (reference)	—	
40–59 years	1.70	1.47, 1.97
≥ 60 years	1.57	1.34, 1.85
**Marital Status**		
Unmarried (reference)	—	
Married	1.22	1.06, 1.40
**Specialty**		
Internal Medicine (reference)	—	
Surgery	1.09	1.02, 1.17
Other Specialty	1.00	0.93, 1.09
**Youngest Child’s Age**		
≤ 5 years (reference)	—	
6–22 years	1.08	0.96, 1.20
≥ 23 years	1.07	0.94, 1.22
No Children	0.96	0.85, 1.09
**Working Hours (hours/day)**	1.07	1.06, 1.09

Estimates are adjusted prevalence ratios (aPRs) from modified Poisson regression with robust (Huber–White) standard errors, adjusted for gender, age group, marital status, medical specialty, youngest child’s age, and working hours. Reference categories are male, age ≤ 39 years, unmarried, and internal medicine. Abbreviations: aPR, adjusted prevalence ratio; CI, confidence interval

### Dual-earner married physicians with children

Patterns mirrored the main analysis: men spent more time in paid work and academic/professional development, whereas women devoted more time to unpaid care and had less leisure on both weekdays and weekends/holidays (Additional files 7, 8, 9).

## Discussion

### Principal findings

In this nationwide sample of full-time hospital physicians, women allocated more time to unpaid care and less to leisure than men on both weekdays and weekends/holidays; on weekdays, they also worked relatively fewer paid hours. As a secondary outcome, women were less likely to report an annual income ≥ ¥15 million (Table 3). Similar patterns in an exploratory subgroup of dual-earner households with children (Additional file 9) suggest that gendered differences in time allocation persist even when both partners are employed.

### Interpretation in context

Our findings align with prior time-use and occupational research showing that women assume more unpaid domestic labor, with gender differences also reported in leisure time and leisure patterns [4, 10]. Because daily time is finite, greater unpaid care for women is associated with less discretionary time and—with smaller magnitude—fewer paid hours. In this framework, increases in one activity necessarily imply reallocations from others, a mechanism that can plausibly limit participation in conference travel, advanced training, and research—activities that accumulate career capital.

#### Workplace structures and culture

While evidence from Japan links fathers’ long work schedules to reduced childcare participation [11], our findings highlight a persistent burden for women: female physicians reported more unpaid care and less leisure than men, despite working only modestly fewer paid hours. This domestic inequality mirrors professional disparities; in surgery, for instance, gender gaps in operative case volume widen over time and are not solely explained by childbearing [12]. Collectively, these patterns suggest that work-style reforms capping paid hours are necessary but insufficient. Without broader cultural and structural changes, institutional limits on hours are unlikely to narrow gender gaps in either unpaid labor or career advancement.

#### Household support and division of labor

Household composition and spousal employment further shape time budgets. Prior work shows that male physicians more often have non-employed spouses, whereas women physicians are commonly in dual-career marriages [13, 14]. Persistence of disparities within our dual-earner subgroup suggests that domestic role expectations—not employment status alone—sustain gendered time use.

### Implications for policy and practice

Hour caps are necessary but not sufficient. Health systems and professional bodies can support more equitable time use by normalizing flexible scheduling options for all physicians and by removing structural disincentives to men’s caregiving (e.g., protected caregiving blocks, equitable on-call swaps). The most immediately modifiable levers lie at the organizational and policy levels. Targeting child care or flexibility to women physicians alone can provide short-term relief, yet it risks entrenching gendered divisions of labor, shifting the equity burden onto women, and potentially widening gaps in discretionary time and advancement. More durable designs are gender-neutral and caregiver-focused; they pair flexibility with incentives that make caregiving feasible for all physicians (e.g., non-birthing parental leave that is practically usable) and include routine, gender-stratified monitoring of uptake and outcomes. Reducing structural barriers to men’s participation in unpaid care work is therefore not only a matter of equity but also a matter of recruitment, retention, workforce sustainability, and organizational quality. Workplaces in which flexibility and caregiving-related leave can be used without strong gendered expectations may be better able to retain physicians of all genders and maintain a healthier organizational climate. A more sustainable and gender-diverse physician workforce may also help health systems better respond to the needs of a diverse patient population. Broader cultural change remains important over the long term; however, employers and policymakers are positioned to act now on the institutional conditions that shape how work and care are allocated.

### Strengths and limitations

Strengths include a large, nationwide sample; the separation of weekday and weekend time use; and multivariable analyses adjusting for major covariates based on a priori conceptual models. These strengths should, however, be interpreted in light of several limitations. First, because this study was based on a nonprobability web panel and the sampling denominator was unavailable, response rates could not be calculated and representativeness of the broader physician population cannot be assured. Participation may have depended on factors such as time availability or interest in work-life balance or gender-related issues, which could have introduced selection bias. The extent or direction of any such bias could not be directly assessed in the present study. Second, the relatively small number of women physicians (316 of 2,540 respondents) may limit more granular gender-stratified analyses. Third, self-reported time-use data may also have introduced measurement uncertainty, recall bias, and social desirability bias. Prior studies suggest that reporting of unpaid care and domestic work may vary by gender and survey method or context, but the direction of any such bias is inconsistent across studies [15–17]. Accordingly, we could not infer the direction of any gender-related reporting bias in the present study. In addition, aggregating activities into whole-hour daily totals clarifies trade-offs between categories but obscures within-day timing and fragmentation of work (e.g., split shifts, overnight calls), which may also have affected daily patterns of time allocation across unpaid care work, leisure, sleep, and other activities. Because daily time use was constrained to 24 h, differences in one activity domain necessarily reflect trade-offs with time available for other domains. The observed gender differences should therefore be interpreted as reallocations within a fixed daily time budget. Fourth, the annual income model adjusted for reported weekday working hours; therefore, the observed gender difference reflects partial adjustment for paid working time. However, this adjustment did not fully capture other time- and compensation-related contributors to annual income, such as weekend/holiday work, overtime compensation, on-call duties, or participation in time-intensive procedures. The adjusted income difference may therefore still reflect unmeasured differences in work patterns and compensation structure.

Finally, the cross-sectional design precludes causal inference and does not allow determination of the temporal ordering between unpaid care work, income, or time-use patterns. Residual confounding from unmeasured factors (e.g., hospital size, hospital function, and workplace-level support for caregiving or work-life balance) also cannot be excluded, although we adjusted for major covariates selected a priori.

### Generalizability

External validity should be considered alongside the study’s limitations. This nationwide sample of full-time hospital physicians supports relevance to Japan’s hospital workforce; however, external validity is constrained by a nonprobability web-panel frame and self-reported time use. Estimates reflect reallocations within a fixed daily time budget and may differ in settings with different scheduling norms, household structures, or policy environments. Nonetheless, the direction and relative magnitudes of gender gaps are consistent with international literature, suggesting cautious transferability to comparable health systems. A priori replication in probability-based samples or administrative time records would strengthen generalizability.

### Future directions

Given these contextual constraints on applicability, several lines of inquiry are warranted. Longitudinal cohorts should test whether shifts in domestic roles precede changes in career outcomes. Future studies could also strengthen causal inference and reduce bias through prospective evaluation of workplace or policy interventions, improved time-use measurement (e.g., time diaries), probability-based sampling where feasible, and analytic approaches that better account for the compositional nature of daily time use. Cross-national comparisons with harmonized indicators (work hours, leave uptake, partner employment) would position Japan within broader patterns. A practical first step is to implement standardized, gender-stratified dashboards at the institutional level using existing HR and scheduling records (e.g., paid hours, on-call frequency, protected academic time, leave uptake). De-identified regional summaries could then be aggregated periodically to evaluate reforms.

## Conclusions

In this cross-sectional sample of hospital physicians in Japan: women reported greater unpaid care work, less discretionary time, and show a lower prevalence of higher income than men even after adjustment. Within a finite 24-hour day, hour caps alone are unlikely to be sufficient; equity efforts should normalize flexible scheduling for all physicians, support men’s caregiving by removing structural disincentives, and embed gender-stratified monitoring.

## Electronic Supplementary Material

Below is the link to the electronic supplementary material.

Supplementary Material 1

Supplementary Material 2

Supplementary Material 3

Supplementary Material 4

Supplementary Material 5

Supplementary Material 6

Supplementary Material 7

Supplementary Material 8

Supplementary Material 9

## Data Availability

Deidentified individual participant data are not available because of contractual restrictions with the survey vendor (M3 Inc.). The survey instrument and analytic code will be made available from the corresponding author upon reasonable request for noncommercial research purposes after publication.

## Abbreviations

AAPOR: American Association for Public Opinion Research
AMD: Adjusted mean difference
aPR: Adjusted prevalence ratio
CI: Confidence interval
