# Supplementary material for: Time use, unpaid care work, and income: a nationwide cross-sectional web survey of gender gaps among hospital physicians in Japan
Source: BMC Health Serv Res. 2026 May 20;26:711. doi: 10.1186/s12913-026-14627-7 (PMC13192210; doi:10.1186/s12913-026-14627-7)
Supplement: Supplementary file 1 — Supplementary Material 1 [file 12913_2026_14627_MOESM1_ESM.docx]

**eAppendix 1. AAPOR Survey Disclosure Statement**

This study adhered to the American Association for Public Opinion Research (AAPOR) Best Practices for Survey Research. Given the operational characteristics of an external physician panel (m3.com), we provide transparent disclosure of the following elements:

- Sampling and recruitment: A nonprobability, volunteer sample of physician members on the m3.com platform. Survey invitations were distributed by m3.com via email on multiple occasions during the field period; participation was voluntary.
- Eligibility and access control: Only authenticated m3.com physician accounts could access the questionnaire. The platform enforced one submission per account (single-response constraint).
- Response rate: A formal AAPOR response rate (RR1–RR6) could not be calculated because the platform did not disclose to investigators the total number of physicians who received or opened the invitations or the number of link click-throughs. Accordingly, no invitation, view, or click denominators were available; we report only the number of completed questionnaires and the field dates.
- Incentives: Completion incentives were provided by the platform as standard m3.com points.
- Questionnaire development: Items were adapted from prior national physician surveys and reviewed internally for clarity. No formal pilot test was conducted before the main fielding.
- Data quality controls: The platform prevented multiple submissions from the same account. We excluded obviously incomplete or inconsistent records according to prespecified criteria. No post-stratification weighting was applied.
- Ethics and consent: Participation was voluntary and anonymous on the platform; completion of the online questionnaire constituted informed consent. Institutional review board approval and data handling procedures are described in the Methods section of the manuscript.

Field period and completed questionnaires: January 9–31, 2024.

Number of completed questionnaires: 3,314.
