# Supplementary material for: Time use, unpaid care work, and income: a nationwide cross-sectional web survey of gender gaps among hospital physicians in Japan
Source: BMC Health Serv Res. 2026 May 20;26:711. doi: 10.1186/s12913-026-14627-7 (PMC13192210; doi:10.1186/s12913-026-14627-7)
Supplement: Supplementary file 2 — Supplementary Material 2 [file 12913_2026_14627_MOESM2_ESM.docx]

**eAppendix 2. Survey Instrument (English Translation)**

This document is an English translation of the Japanese survey instrument used in this study.

**Q1. Participation in the survey**

Participation in this survey is voluntary. By completing the questionnaire, you are indicating your informed consent to participate. If you wish to later withdraw your consent, please contact M3 Inc.

(Please check the box to indicate your agreement to participate.)

**Q2-1. What is your gender?**

Male

Female

Other (please specify): ______

**Q2-2. Please enter your age**

[Free text]

**Q3. What is your primary occupation?**

**Please select the one that best describes your current position.**

**(“Full-time” refers to individuals who work all hours specified by the institution. Those working fewer than 32 hours per week are considered part-time workers.)**

Full-time physician

Part-time physician

Private practice doctor (clinic or private facility)

Hospital staff

Employee of a pharmaceutical or medical device company

University faculty (assistant professor or above)

University hospital staff (including residents and other medical professionals)

Other

**Q4. What is your primary specialty?**

Internal Medicine and Subspecialties

- Cardiovascular medicine

- Pulmonary medicine

- Gastroenterology

- Endocrinology

- Neurology

- Other internal medicine subspecialties (please specify): ______

Surgery and Related Fields

- General, cardiovascular, thoracic, gastrointestinal, neurosurgical, and pediatric surgeries

- Orthopedic surgery

- Breast surgery

- Plastic and reconstructive surgery

- Obstetrics and gynecology

- Urology

- Ophthalmology

- Otolaryngology

- Other surgical subspecialties (please specify): ______

Other

- Dermatology

- Pediatrics

- Psychiatry

- Anesthesiology

- Radiology

- Resident

- Other

**Q5. What is your current working status?**

**Please select the option that best describes your situation during the survey period.**

Currently on maternity or paternity leave

Currently on childcare leave

Currently taking paid leave for childcare, totaling 14 or more calendar days (including at least 10 weekdays)

Currently on 14 or more calendar days (including at least 10 weekdays) of paid leave or absence for reasons other than childcare (e.g., caregiving, personal illness)

None of the above (working as usual)

**Q6-1. What is your annual household income?**

Less than 5 million yen

5 to less than 10 million yen

10 to less than 15 million yen

15 to less than 20 million yen

20 to less than 25 million yen

25 to less than 30 million yen

30 million yen or more

**Q6-2. What is your own annual income?**

Less than 5 million yen

5 to less than 10 million yen

10 to less than 15 million yen

15 to less than 20 million yen

20 to less than 25 million yen

25 to less than 30 million yen

30 million yen or more

**Q7. Do you have a spouse or partner?**

**(This includes both legal spouses and long-term cohabiting partners, regardless of gender.)**

Single (never married)

Single (divorced or widowed)

Married or partnered (living together)

Married or partnered (living separately)

**Q8. What is your spouse’s or partner’s occupation?**

Hospital-based doctor

Private practice doctor (clinic or private facility)

Healthcare professional (excluding doctors)

Company Employee (e.g., office worker, corporate staff)

Self-employed

Full-time homemaker

Not currently working

Other

**Q9. Do you have any children? (regardless of whether they live with you)**

No children

1 child

2 children

3 or more children

**Q10. Of the children living with you, how old is your youngest child?**

(If none of your children live with you, please leave this blank.)

[Free text: Age in years]

**Q11. Please indicate your average weekday time use over the past month**

**(Note: Total must equal 24 hours. All responses in whole hours.)**

Working hours: __ hours

Commuting time: __ hours

Self-study time: __ hours

Academic activity time: __ hours

Unpaid care work (housework, childcare): __ hours

Meals and personal care time: __ hours

Leisure time: __ hours

Sleeping time: __ hours

Other: __ hours

Total: 24 hours

**Q12. Please indicate your average weekend and holiday time use over the past month**

**(Total must equal 24 hours. All responses in whole hours.)**

Working hours: __ hours

Commuting time: __ hours

Self-study time: __ hours

Academic activity time: __ hours

Unpaid care work (housework, childcare): __ hours

Meals and personal care time: __ hours

Leisure time: __ hours

Sleeping time: __ hours

Other: __ hours

Total: 24 hours

**Q13-1. For each of the following statements (1)–(5), please indicate your level of agreement**

(1) It is desirable for men and women to share housework and childcare equally.

(2) Men should work outside the home, while women should do housework and childcare.

(3) Since women may leave the workplace to give birth and raise children, it is justifiable to limit their admission to medical school.

(4) Protesting against various types of harassment is not always desired.

(5) I believe it is unfair that female doctors are allowed to take excessive advantage of childcare benefits.

Response options:

- Strongly agree

- Agree

- Disagree

- Strongly disagree

**Q14. Please use the box below to comment on any aspect of the survey’s content or design.**

[Free response]
