# Supplementary material for: Time use, unpaid care work, and income: a nationwide cross-sectional web survey of gender gaps among hospital physicians in Japan
Source: BMC Health Serv Res. 2026 May 20;26:711. doi: 10.1186/s12913-026-14627-7 (PMC13192210; doi:10.1186/s12913-026-14627-7)
Supplement: Supplementary file 3 — Supplementary Material 3 [file 12913_2026_14627_MOESM3_ESM.docx]

**Supplemental Table 1. Unadjusted time-use summaries by gender (hours/day)**

|  | **Weekdays** | | | **Weekends/holidays** | | |
| --- | --- | --- | --- | --- | --- | --- |
| Activity category | Men, Mean [SD] (hours) | Women, Mean [SD]  (hours) | P value | Men, Mean [SD] (hours) | Women, Mean [SD] (hours) | P value |
| **Working hours** | 9.12 [1.93] | 8.45 [1.96] | < 0.001 | 2.73 [3.47] | 2.32 [3.36] | 0.045 |
| **Academic & professional development** | 1.82 [1.44] | 1.57 [1.23] | < 0.001 | 2.15 [2.19] | 1.80 [1.83] | <0.001 |
| **Commuting** | 1.33 [0.79] | 1.32 [0.68] | 0.803 | 0.65 [0.84] | 0.50 [0.81] | 0.002 |
| **Unpaid care work** | 1.01 [1.11] | 2.56 [1.98] | <0.001 | 2.10 [2.64] | 4.44 [3.76] | < 0.001 |
| **Meals & personal care** | 1.78 [1.89] | 1.58[1.61] | 0.049 | 2.82 [3.02] | 2.63 [2.47] | 0.21 |
| **Leisure** | 2.43 [1.75] | 1.98 [1.74] | < 0.001 | 6.50 [4.29] | 4.95 [3.85] | < 0.001 |
| **Sleeping** | 6.51 [1.18] | 6.54 [1.13] | 0.657 | 7.06 [1.28] | 7.37 [1.35] | < 0.001 |

All units are hours per day. Values represent unadjusted means [standard deviations] of self-reported time use for each activity category. Abbreviations: SD, standard deviation
