# Supplementary material for: Time use, unpaid care work, and income: a nationwide cross-sectional web survey of gender gaps among hospital physicians in Japan
Source: BMC Health Serv Res. 2026 May 20;26:711. doi: 10.1186/s12913-026-14627-7 (PMC13192210; doi:10.1186/s12913-026-14627-7)
Supplement: Supplementary file 4 — Supplementary Material 4 [file 12913_2026_14627_MOESM4_ESM.docx]

**Supplemental Table 2. Weekdays—multivariable-adjusted gender differences in time use (female − male), hours/day**

| Activity category | **Adjusted mean difference, hours/day (female − male)** | **95% CI** |
| --- | --- | --- |
| **Working hours** | –0.82 | –1.06, –0.59 |
| **Academic & professional development** | –0.34 | –0.51, –0.18 |
| **Commuting** | 0.05 | –0.05, 0.15 |
| **Unpaid care work** | 1.51 | 1.31, 1.70 |
| **Meals & personal care** | –0.33 | –0.55, –0.11 |
| **Leisure** | –0.78 | –0.97, –0.60 |
| **Sleeping** | –0.10 | –0.24, 0.04 |

Adjusted mean differences (female − male) in hours/day with 95% CIs across activity categories, estimated using multivariable linear regression adjusted for age, marital status, youngest child’s age, specialty, and weekly working hours (weekly working hours omitted when modeling working hours). Positive values indicate more time for women; negative values indicate more time for men. Abbreviations: CI, confidence interval.
