# Supplementary material for: Time use, unpaid care work, and income: a nationwide cross-sectional web survey of gender gaps among hospital physicians in Japan
Source: BMC Health Serv Res. 2026 May 20;26:711. doi: 10.1186/s12913-026-14627-7 (PMC13192210; doi:10.1186/s12913-026-14627-7)
Supplement: Supplementary file 5 — Supplementary Material 5 [file 12913_2026_14627_MOESM5_ESM.docx]

**Supplemental Table 3.** **Weekends/holidays—multivariable-adjusted gender differences in time use (female − male), hours/day**

| Activity category | **Adjusted mean difference, hours/day (female − male)** | **95% CI** |
| --- | --- | --- |
| **Working hours** | –0.70 | –1.11, –0.28 |
| **Academic & professional development** | –0.43 | –0.67, –0.19 |
| **Commuting** | –0.10 | –0.19, –0.02 |
| **Unpaid care work** | 2.35 | 1.98, 2.72 |
| **Meals & personal care** | –0.09 | –0.45, 0.26 |
| **Leisure** | –1.90 | –2.30, –1.50 |
| **Sleeping** | 0.16 | 0.01, 0.32 |

Adjusted mean differences (female − male) in hours/day with 95% CIs across activity categories, estimated using the same multivariable linear regression models and covariate adjustments as Supplemental Table 2. Positive values indicate more time for women; negative values indicate more time for men. Abbreviations: CI, confidence interval.
