# Supplementary material for: Time use, unpaid care work, and income: a nationwide cross-sectional web survey of gender gaps among hospital physicians in Japan
Source: BMC Health Serv Res. 2026 May 20;26:711. doi: 10.1186/s12913-026-14627-7 (PMC13192210; doi:10.1186/s12913-026-14627-7)
Supplement: Supplementary file 6 — Supplementary Material 6 [file 12913_2026_14627_MOESM6_ESM.docx]

**Supplemental Table 4. Sensitivity analyses excluding marital status and child-related variables from models of unpaid care work on weekdays and weekends/holidays**

| **Outcome** | **Main model: sex coefficient** | **SE** | **95% CI** | **P value** | **Sensitivity model: sex coefficient** | **SE** | **95% CI** | **P value** |
| --- | --- | --- | --- | --- | --- | --- | --- | --- |
| **Weekday** | –0.78 | 0.10 | –0.98, –0.58 | < 0.001 | –0.59 | 0.10 | –0.78, –0.39 | < 0.001 |
| **Weekend/holiday** | –1.89 | 0.22 | –2.32, –1.46 | < 0.001 | –1.56 | 0.22 | –1.99, –1.13 | < 0.001 |

Abbreviations: SE, standard error; CI, confidence interval.

Main models were adjusted for age category, specialty, marital status, youngest child’s age, and working hours. Sensitivity models excluded marital status and child-related variables from the adjustment set. Negative coefficients reflect the coding of the sex variable in the regression model; the direction and statistical significance of the association were unchanged across models.
