# Supplementary material for: Time use, unpaid care work, and income: a nationwide cross-sectional web survey of gender gaps among hospital physicians in Japan
Source: BMC Health Serv Res. 2026 May 20;26:711. doi: 10.1186/s12913-026-14627-7 (PMC13192210; doi:10.1186/s12913-026-14627-7)
Supplement: Supplementary file 7 — Supplementary Material 7 [file 12913_2026_14627_MOESM7_ESM.docx]

**Supplemental Table 5.** Subgroup (dual-earner parents): Weekdays and Weekends/holidays time-use differences by gender

|  | Weekdays | | | Weekends/holidays | | |
| --- | --- | --- | --- | --- | --- | --- |
| Activity category | Men, Mean [SD] (hours) | Women, Mean [SD]  (hours) | P value | Men, Mean [SD] (hours) | Women, Mean [SD] (hours) | P value |
| Working hours | 9.27 [1.88] | 8.02 [2.12] | < 0.001 | 3.06 [3.63] | 2.66 [3.52] | 0.289 |
| Academic & professional development | 1.88 [1.88] | 1.28 [2.12] | < 0.001 | 2.01 [1.99] | 1.50 [1.65] | 0.006 |
| Commuting | 1.30[0.72] | 1.29 [0.63] | 0.859 | 0.75 [0.86] | 0.45 [0.59] | < 0.001 |
| Unpaid care work | 1.46 [1.29] | 4.25 [2.08] | < 0.001 | 3.30 [3.23] | 7.37 [4.27] | < 0.001 |
| Meals & personal care | 1.55 [1.91] | 1.44 [1.49] | 0.500 | 2.42 [2.89] | 2.01 [2.04] | 0.079 |
| Leisure | 2.11 [1.62] | 1.11 [1.11] | < 0.001 | 5.57 [3.91] | 2.93 [2.88] | < 0.001 |
| Sleeping | 6.43 [1.27] | 6.61 [1.11] | 0.144 | 6.88 [1.39] | 7.08 [1.17] | 0.123 |

**Footnote:** All units are hours per day. Values are unadjusted means [standard deviations]. P values are from 2-sided tests comparing men and women within each day type (weekdays and weekends/holidays). No multiplicity adjustment was applied as this is an exploratory subgroup analysis. Abbreviations: SD, standard deviation.
