# Supplementary material for: Time use, unpaid care work, and income: a nationwide cross-sectional web survey of gender gaps among hospital physicians in Japan
Source: BMC Health Serv Res. 2026 May 20;26:711. doi: 10.1186/s12913-026-14627-7 (PMC13192210; doi:10.1186/s12913-026-14627-7)
Supplement: Supplementary file 8 — Supplementary Material 8 [file 12913_2026_14627_MOESM8_ESM.docx]

**** **Supplemental Figure 1. Subgroup (dual-earner parents): weekday time-use differences by gender**

Points show adjusted mean differences (female − male) in hours/day with 95% confidence intervals (CIs) among legally married physicians with children whose partners are employed. Models adjusted for age, marital status, youngest child’s age, specialty, and working hours (working hours omitted when modeling working hours).
