# Supplementary material for: Time use, unpaid care work, and income: a nationwide cross-sectional web survey of gender gaps among hospital physicians in Japan
Source: BMC Health Serv Res. 2026 May 20;26:711. doi: 10.1186/s12913-026-14627-7 (PMC13192210; doi:10.1186/s12913-026-14627-7)
Supplement: Supplementary file 9 — Supplementary Material 9 [file 12913_2026_14627_MOESM9_ESM.docx]

 **Supplemental Figure 2. Subgroup (dual-earner parents): weekend/holiday time-use differences by gender**

Points show adjusted mean differences (female − male) in hours/day with 95% confidence intervals (CIs) among legally married physicians with children whose partners are employed, assessed on weekends/holidays. Models were adjusted for age, marital status, youngest child’s age, specialty, and working hours (working hours omitted when modeling working hours).
